# Tailoring mechanical properties and degradation rate of maxillofacial implant based on sago starch/polylactid acid blend

**DOI:** 10.1016/j.heliyon.2021.e08600

**Published:** 2021-12-13

**Authors:** Y. Whulanza, A. Azadi, S. Supriadi, S.F. Rahman, M. Chalid, M. Irsyad, M.H. Nadhif, P. Kreshanti

**Affiliations:** a Department of Mechanical Engineering, Faculty of Engineering, Universitas Indonesia, Indonesia; b Research Center on Biomedical Engineering, Universitas Indonesia, Indonesia; c Indonesian Agency for Agricultural Research and Development, Indonesia; d Department of Electrical Engineering, Faculty of Engineering, Universitas Indonesia, Indonesia; e Department of Metallurgical and Material Engineering, Faculty of Engineering, Universitas Indonesia, Indonesia; f Medical Technology Cluster, Indonesian Medical Education and Research Institute (IMERI), Faculty of Medicine, Universitas Indonesia, Indonesia; g Department of Medical Physics, Faculty of Medicine, Universitas Indonesia, Indonesia; h Plastic Reconstructive and Aesthetic Surgery Division, Department of Surgery, Cipto Mangunkusumo Hospital, Faculty of Medicine, Universitas Indonesia, Indonesia

**Keywords:** Degradable implant, Maxillofacial fracture, Fast implant degradation, Pediatric patients, Sago starch/polylactic acid/polyethylene glycol blend, Finite element analysis

## Abstract

A polymeric bone implants have a distinctive advantage compared to metal implants due to their degradability in the local bone host. The usage of degradable implant prevents the need for an implant removal surgery especially if they fixated in challenging position such as maxillofacial area. Additionally, this fixation system has been widely applied in fixing maxillofacial fracture in child patients. An ideal degradable implant has a considerable mass degradation rate that proved structural integrity to the healing bone. At this moment, poly(lactic acid) (PLA) or poly(lactic-co-glycolic acid) (PLGA) are the most common materials used as degradable implant. This composition of materials has a degradation rate of more than a year. A long degradation rate increases the long-term biohazard risk for the bone host. Therefore, a faster degradation rate with adequate strength of implant is the focal point of this research. This study tailored the tunable degradability of starch with strength properties of PLA. Blending system of starch and PLA has been reported widely, but none of them were aimed to be utilized as medical implant. Here, various concentrations of sago starch/PLA and Polyethylene glycol (PEG) were composed to meet the requirement of maxillofacial miniplate implant. The implant was realized using an injection molding process to have a six-hole-miniplate with 1.2 mm thick and 34 mm length. The specimens were physiochemically characterized through X-ray diffraction, differential scanning calorimetry, thermogravimetric analysis, and Fourier Transform Infrared spectroscopy. It is found that the microstructure and chemical interactions of the starch/PLA/PEG polymers are correlated with the mechanical characteristics of the blends. Compared to a pure PLA miniplate, the sago starch/PLA/PEG blend shows a 60–80% lower tensile strength and stiffness. However, the flexural strength and elongation break are improved. A degradation study was conducted to observe the mass degradation rate of miniplate for 10 weeks duration. It is found that a maximum concentration of 20% sago starch and 10% of PEG in the PLA blending has promising properties as desired. The blends showed a 100–150% higher degradability rate compared to the pure PLA or a commercial miniplate. The numerical simulation was conducted and confirmed that the miniplate in the mandibular area were shown to be endurable with standard applied loading. The mechanical properties resulted from the experimental work was applied in the Finite Element Analysis to find that our miniplate were in acceptable level. Lastly, the in-vitro test showed that implants are safe to human cell with viability more than 80%. These findings shall support the use of this miniplate in rehabilitating mandibular fractures with faster degradation with acceptance level of mechanical characteristic specifically in case of 4–6 weeks bone union.

## 1 Introduction

Implant fixation, using screws and bone plates, is a type of medical process used to replace the structure and functionality of a biological part. Plates are used to join bones from two or more tips of fracture fragments [1]. Biodegradable screws and plates have been used to fix bone in the past three decades [2, 3, 4, 5, 6], and especially maxillofacial bone. Biodegradable implants provide some advantages compared with metallic implants as they prevent the need for implant removal surgery because they are biodegradable and do not hinder the rapid growth of the original bone for patients who are still in growth period [7, 8, 9, 10]. Specifically, during the fixation, less bone moves, such as maxillofacial bone. A degradable fixation system also reduces the long-term biohazard risk for the bone host, such as corrosion, osteoporosis caused by stress shielding, and the negative effects from electro-medical devices, i.e., computer tomography scanning (CT), radiography, and magnetic resonance imaging [10, 11]. However, some problems might occur with the use of these devices, such as the implant's tensile strength decreasing too fast and a mismatch of the degradability rate between the growth of host bone and the implant system. The implant should degrade by the time the fracture regains its structural integrity due to its recovery growth [12] since the bone remodeling could occur in 3 months to 2 years [13]. However, a newer study stated that the bone remodeling could start at the 4^th^ week after fracture and continue after the 8^th^ week in a normal case of fracture [14]. Whereas, existing miniplates took 12 months to 5 years to degrade after implanted [9]. Therefore, tuning the degradability rate of a miniplate fabricated through injection molding with a blending system of synthetic and natural materials is of interest.

Bone fixation systems composed of biodegradable polymers, such as poly(lactic acid) (PLA) or poly(lactic-co-glycolic acid) (PLGA), have been thoroughly investigated as they have an elasticity modulus similar to those of skeletal tissues. The elasticity modulus of a biodegradable polymer such as PLA is sufficient to decrease the stress shielding between the implant and bones [15, 16]. However, PLA has a slow degradation period of about 2–5 years in the crystalline phase, which is due to its hydrophobicity and being resistant to the hydrolysis process in the biological system. A similar characteristic was observed for PLGA, which can cause several tissue complications [9, 17]. Therefore, research on new materials has been conducted by combining synthetic and natural polymers. This compound must have the mechanical characteristics of synthetic polymers as well as better degradability than natural polymers [18, 19, 20].

Starch-based polymers have been shown to have potential for bone implants because of their biocompatibility and biodegradability [21, 22, 23, 24, 25]. Starch is a hydrophilic polymer and is sensitive to water, enabling the adjustment of its degradability characteristics. However, the mechanical characteristics of the blend are determined by the starch content [26]. Generally, starch with PLA blending shows weak interactions at certain concentration [27, 28]. Therefore, the addition of an extra substance, like a compatibilizer agent, is expected to increase the compatibility of starch/PLA [29, 30]. Polyethylene glycol (PEG) has good solubility in water and organic solvents [31], and thus contributes to modifying starch/PLA compounds. PEG also decreases the glass transition temperature, which benefits material processing, such as mechanical mixing or injection molding. An optimum proportion of PEG needs to be used so that it does not compromise the mechanical characteristics of the final product [32, 33].

Sago starch (*Metroxylon sago*) is abundantly and natively found in Southeast Asia and Oceania [34, 35]. Starch is composed of two important fractions, amylose, which is a linear polymer, and amylopectin, which is a highly branched polymer of α-glucose units. Sago starch has been found to have a higher amylose content than corn and potato starches [36]. A high amylose content tends to form a crystalline polymer, which produces stronger mechanical properties than amorphous amylopectin [37, 38]. Therefore, being an abundant natural resource with high compatibility with human tissues makes sago starch a strong candidate for producing the desired blending composition [39, 40].

Injection molding systems have been widely used in the manufacture of products derived from thermoplastic materials with complex shapes and precise dimensions. The system has also been introduced the mixing of PLA and starch blending in medical application [41]. It was reported that the mechanical properties of PLA material can be improved by manipulating the thermochemical properties during the injection molding process. The report showed a shifting in the mechanical properties of PLA occurred during the melting process with an increase in melting temperature and mold temperature [41]. Recently, fabrication of medical plates for maxillofacial with PLGA material using the injection molding method has been studied by Melo et al. [42].

Our study showed that PLA blends used for various biomedical applications have been physically and chemically characterized [42, 43]. However, no studies have been published on fracture fixation miniplates based on sago starch/PLA in combination with PEG. In this paper, an exploration in sago starch/PLA/PEG compound fabricated as a miniplate product was thoroughly observed. The physicochemical properties of the compound were acquired from X-ray diffraction, differential scanning calorimetry, thermogravimetric analysis, and Fourier Transform Infrared spectroscopy. The mechanical properties reported here includes tensile and bending test that reveals the strength, elastic modulus, and elongation at break of miniplate. A correlation of mechanical properties and physicochemical properties shall be reflected in the degradation rate and closely examined by the Scanning Electron Microscopy observation. Ultimately, a finite element study was conducted at our optimal compound to simulate a scenario in a mandibular fracture fixation. An optimized blending system must withstand a standard post-operative loading force. Moreover, the miniplate shall has faster degradation compared to that available product in 4–6 weeks observation.

## 2 Materials and methods

### 2.1 Materials

Polylactic acid (PLA) was supplied by Zhuhai Sunlu Industrial Co. Ltd. (Shanghai, China) with melting point of 155°С and molecular weight of 160,000 g/mol. The sago starch (*Metroxylon sago*) with a 27% amylose content was produced by PT ANJAP (Papua, Indonesia). PEG 4000, as a plasticizer with a molecular weight of 4,000 g/mol and density of 1.2 g/cm^3^, was produced by Merck (Darmstadt, Germany). Methylene chloride (MC), used as a solvent, and phosphate-buffered saline (PBS), pH of 7.3 for the biodegradability test on miniplate products, were purchased from Sigma-Aldrich (Darmstadt, Germany).

### 2.2 Blend preparation

Initially, two blend formulations were prepared: a sago starch/PLA blend and a PLA/PEG blend. Later, the sago starch/PLA was mixed with a PLA/PEG compound to form the final sago starch/PLA/PEG compound. The detail steps are explained below.

Firstly, PLA pellets and sago starch were mixed in various concentrations in a solvent of dichloromethane (methylene chloride (MC)) to form a sago starch/PLA blend (Table 1) [44,45]. The solution [21, 35] was then stirred at 600 rpm for 40 min. The blend was then cast on a glass baking tray and dried in an oven at 40 °C for 20 h. The blending was cut into pieces to be prepared in an extruder machine [23, 46, 47].

**Table 1:** Composition of miniplate specimens: sago starch/PLA, PLA/PEG, and sago starch/PLA/PEG blend formulations. PLA, polylactic acid; PEG, polyethylene glycol.

Specimen Code	Chemical Compounds	Weight Fraction of Sago Starch	Weight Fraction of PLA	Weight Fraction of PEG
PLA	Pure PLA	0	100	0
SPLA10	Sago Starch/PLA	10	90	0
SPLA20	Sago Starch/PLA	20	80	0
SPLA30	Sago Starch/PLA	30	70	0
SPLA40	Sago Starch/PLA	40	60	0
SPLA50	Sago Starch/PLA	50	50	0
PLAP10	PLA/PEG	0	90	10
SPLA20P10	Sago Starch/PLA/PEG	SPLA20/PEG: 90/10		
SPLA20P20	Sago Starch/PLA/PEG	SPLA20/PEG: 80/20		

Secondly, a blend of PLA with PEG plasticizer without sago starch was prepared with a weight ratio of 90:10. The two compounds were mixed in a mixer (Brabender GmbH, Duisburg, Germany) at 160 °C at 100 rpm for 10 min. The blend was then mixed with 20% sago starch/PLA (SPLA20) in a ratio of 9:1 with an 8:2 weight fraction. The blending was carried out in an extruder from Haake Rheomix (Waltham, MA, USA). The temperature was set to 180 °C. The extrudate was then dried, comminuted, and stored in a desiccator for 20 h.

### 2.3 Production of miniplates

**Figure 1:**
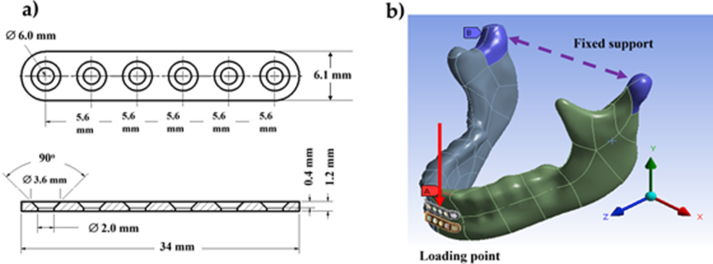
Schematic representation of: (**a**) top and peripheral views of the six-holes-miniplate with the designed dimensions and (**b**) arrangement of two parallel miniplates fixated on a human mandibular model with the red line indicating a simulated loading point on the halves of mandibular.

The sago starch/PLA/PEG compound was processed at 160 °C in an injection molding machine (ELITE E-80B, Elite Industrial Holding, Shenzhen, China) with a holding pressure at 500 MPa, cooling time 24 s, and molding temperature of 30 °C. The mold was previously designed to produce a six-hole miniplate that is 1.2 mm thick, 6.1 mm wide, and 34.0 mm long. A three-dimensional model of the miniplate was built using Autodesk Inventor (Figure 1a).

### 2.4 Finite element analysis (FEA)

A finite element analysis (FEA) software has an advantage to give better and quick visualization behind a mathematical formula. In the FEA study, the specimen was assumed as an isotropic and compressible neo-Hookean material. These set assumptions might result a deviation between the numerical study and the experimental result. Therefore, a verification step was taken by conducting FEA study to simulate the deformation process of specimen by applying force equivalent in the experimental setup. A grid independence test was also conducted to verify the mesh size that appropriate to our simulation. Initially, a various mesh size was arranged at 0.2 mm–2 mm, which will be selected once the mesh convergence was completed.

A 3D model of mandible data was purchased from Synbone AG (Zizers, Switzerland) in STL format. The data were imported to an Autodesk Inventor 3D modeling software and then modified to create a jawbone split into two parts (Figure 1b). Afterwards, the resulted designs were computationally analyzed using finite element analysis (FEA). In the FEA environment, the miniplates were connected to the mandible using mini screws. Two parallel miniplates were fixed to cover the fracture line and the halves of the mandible [48]. The FEA of the mandible used a static structural mechanics module in an ANSYS simulation software [49, 50]. A 50 N force was applied to the maxillofacial miniplates located at the superior and inferior mandible (red line) [51], whereas the fixed support was placed at molar region (purple dashes), shown in Figure 1b.

The mechanical properties of the miniplate were assigned upon the completion of mechanical characterization of the sago starch/PLA/PEG. The successful scenario of the implants was indicated by the values of peak von Mises stress (PVMS) resulted by the working forces on both superior and inferior plates. The VMS is a theoretical stress at a specific finite element of a material to indicate the resulted forces applied at the element from three different axes. The peak VMS (PVMS) is obtained from the finite element that receives the highest value of VMS in a material. The PVMS below the tensile strength of the composites indicates that the material succeeds to retain the working forces, and vice versa. A complete setup of mechanical properties and boundary setting was detailed in Table 2.

**Table 2:** Boundary settings and mechanical properties used in FEA.

Feature	Description		
**Material**		Elastic modulus	Poisson ratio
	Cortical bone	15,000 [11]	0.33 [11]
	Cancellous bone	1,500 [11]	0.30 [11]
	Miniplate	Following the result of this study	0.46 [11]
**Contact connection**	Mandible-screw	No separation	
	Mandible-screw	Bonded	
	Mandible-miniplate	No separation	
	Miniplate-screw	No separation	

### 2.5 TGA and DSC analysis

The analysis was performed on a simultaneous thermal analyzer (Labsys Evo 1600 Setaram Instrumentation, Caluire, France). Thermogravimetric analysis (TGA) was used to describe the thermal stability of the miniplate. Each sample (20 mg) was heated from 25 to 600 °C at a heating rate of 20 °C/min under an argon atmosphere. The mass change data were analyzed to estimate the temperature of the onset of degradation (T_onset_) of the composite.

The thermal transition of the compound was investigated by placing the sample (15–20 mg) on an aluminum pan that was initially heated from 0 to 200 °C at a heating rate of 10 °C/min. Data output determined the glass transition temperature (T_g_), cold crystallization temperature (T_cc_), and melt temperature peak (T_m_). The enthalpy of fusion (ΔH_m_) and enthalpy of crystallization (ΔH_cc_) were also gathered from this measurement. The melting properties were acquired during the heating scan, whereas the crystallization properties were acquired during the cooling scan.

### 2.6 Fourier Transform Infrared red spectroscopy (FTIR)

The FTIR spectra were recorded on a Fourier Transform Infra-Red Nicolet™ iS50 Spectrometer (Thermo Fisher Scientific, Waltham, USA) in the spectral range of 15–27,000 cm^−1^, operated using OMNIC software. The result of transmittance was compared with literature values to determine its functional groups.

### 2.7 X-ray diffraction (XRD) analysis

The microcrystalline structure was determined using X-ray diffraction at room temperature. Samples were characterized using XRD Panalytical Empyrean (Malvern Panalytical, Malvern, UK) at 40 kV and 30 mA with Cu anode radiation at wavelength of 1.5406 Å. The result analyzed using HighScore Plus version 3.0 Standpoint with 2θ between 10° and 80° with a 0.0263°/step scanning rate. Crystallinity degree (X_c_) was calculated using:

(1) X_c_ (%) = Area of crystalline peaks/Area of all peaks × 100%.

### 2.8 Scanning Electron Microscopy (SEM)

The micromorphology of the miniplate was observed with SEM (FEI Quanta 650) with an acceleration voltage of 7.5 kV. Specimens were prepared under liquid nitrogen and coated with gold before observing the surface fracture. The magnification was arranged at 1,000 times to observe the sago starch blending with PLA and PEG.

### 2.9 Mechanical properties

Five miniplates of each composite was prepared before the mechanical testing, which used an MCT-2150 A&D Company (Tokyo, Japan) universal testing machine (UTM) with a maximum force of 500 N at a crosshead speed of 10 mm/min. The tensile test that was based on the ASTM D638 Standard [52] was performed to determine the tensile strength (TS), Young's modulus (E), and elongation at break (EB). Meanwhile, the flexural test was conducted based on the ASTM D790 Standard [53] to determine the flexural strength, flexural modulus, and elongation at break. As a control, a commercial miniplate, Inion® (Inion Oy, Tampere, Finland), was also tested under similar standards.

### 2.10 Biodegradation test

The biodegradation test miniplate was performed according to ISO 10993-5 (E): Biological evaluation of medical devices, Part 5: Tests for in vitro cytotoxicity [54, 55]. Miniplate samples were immersed in a falcon tube with 20 mL of phosphate-buffered saline (PBS) at pH 7.3 ± 0.2 and then closed tightly. Samples were stored in a thermostatic bath with a stable temperature of 37 ± 1 °C for 7, 14, 21, and 28 days. At each time point, the PBS solution pH was checked, and the samples were removed from the solution then dried at 90 °C for 30 min until constant weight. Measurements of miniplate loss were calculated as:

(2) W_loss_ (%) = m_d_/m_o_ × 100%,

where W_loss_ (%) is the average degradation rate, mo is initial weight, and md is final weight. However, the result of the degradation mass was showed as remaining weight which calculated as:

(3) Remaining weight (%) = 100 – W_loss_,

### 2.11 Biocompatibility test

Biocompatibility test of miniplates were conducted using a 3-(4,5-dimethylthiazol-2-yl)-5-(3-carboxymethoxyphenyl)-2-(4-sulfophenyl)-2H-tetrazolium Cell Proliferation (MTT) Assay Kit from Promega (Madison, USA) [56, 57, 58]. Firstly, the adipose-derived human mesenchymal stem cells from ATCC (Manassas, USA) were cultured in RPMI 1640 medium from Biowest (Nualle, France) with 10% FBS, 1% antibiotic-antimycotic, 1% amphotericin B, 1% nanomycopulitine (all from Biowest, Nualle, France) and 0.1 % gentamicin (Gibco, Waltham, USA). This medium was user for all miniplate's viability test. Cells and medium without a miniplate specimen were observed as the control.

Cell viability measurements were carried out at 24, 48 and 36 h after seeding. The previous cell growth medium was discarded and replaced with 450 μl of new medium in each well. A 50 μl of MTS was added, then incubated for 3 h in a 5% CO2 incubator at 37 °C. The medium and MTS were then collected and transferred to 96 well plates (100 μl each with 4 repetitions). The absorbance of the medium and MTS was measured at a wavelength of 490 nm using Multiskan GO spectrophotometer (Thermo Scientific, Waltham, USA). The cell viability was quantified as the percentage relative of absorbance of the resulting solution compared to the control at 24 h after seeding.

Cell suspensions were diluted twofold with a trypan blue solution 0.4% (w/v) (Bio-Rad, California, USA). Ten μL of the mixture was placed on a Bright-Line hemocytometer glass (Sigma-Aldrich, Steinheim, Germany), and analyzed by using a light microscope (Eclipse Ti–S inverted, Nikon Instruments, New York, USA) with 10× magnification. Cells were counted in 25 frames of the hemocytometer according to the manufacturer's recommendations. The percentage of cell viability calculated based on the number of living cells compared to the control.

## 3 Results and discussion

**Figure 2:**
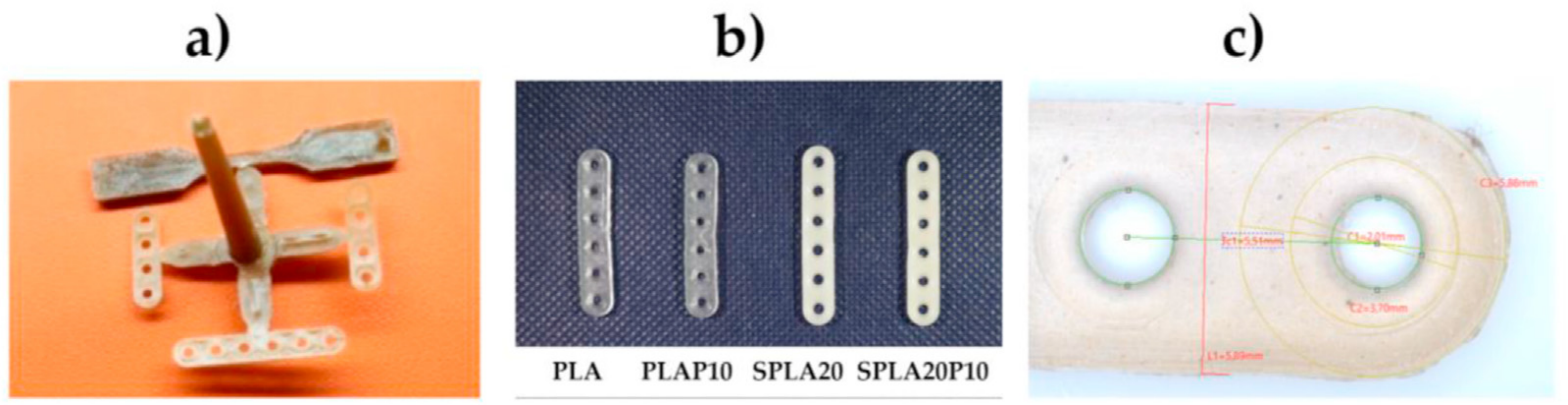
Miniplate specimens: (**a**) the product of injection molding with a gate system; (**b**) six-hole miniplate with different material blending systems: PLA/PEG, sago starch/PLA, and sago starch/PLA/PEG; (**c**) a detailed photograph of the miniplate specimen used to observe the dimensional accuracy.

Figure 2 depicts the miniplate samples from various compounds: pure PLA, PLA/PEG blend, and sago starch/PLA/PEG blend. The miniplate was produced using injection molding process with a premixing of two blending system: starch/PLA and PLA/PEG compound. The pure PLA and PLA/PEG had a clear and transparent color, whereas the sago starch/PLA had a yellow to brownish color depending on the sago starch concentration. The sample was designed to have six holes fixed with a 2 mm diameter screw.

### 3.1 Miniplate morphology

**Figure 3:**
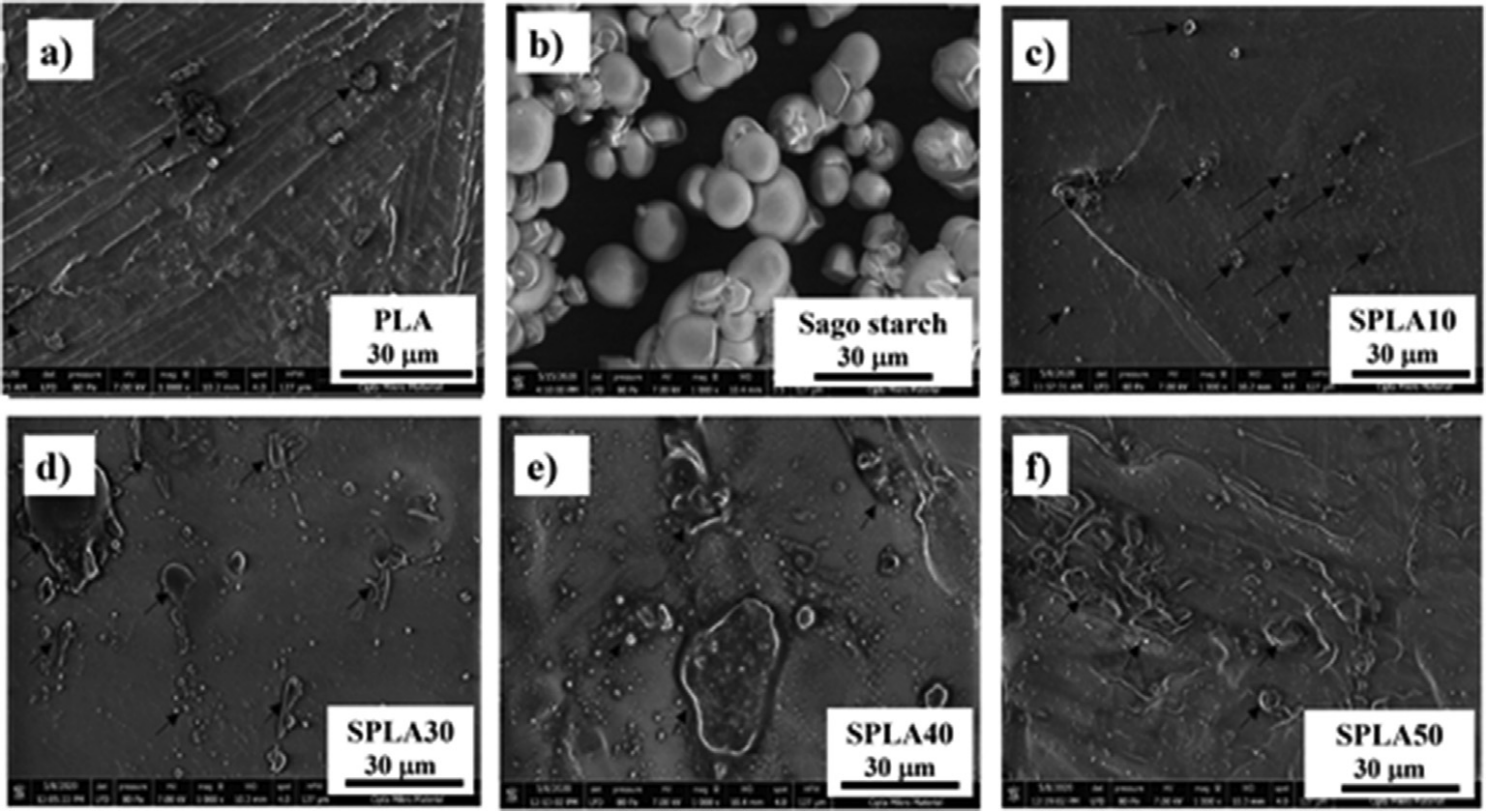
Scanning electron microscopy (SEM) images of the miniplate surface of: (**a**) pure PLA; (**b**) pure sago starch; (**c**) 10% sago starch/PLA; (**d**) 30% sago starch/PLA; (**e**) 40% sago starch/PLA; (**f**) 50% sago starch/PLA.

The microstructure of the pure PLA miniplate presented a surface with unidirectional flow resulting from the injection molding process (Figure 3a). The sago starch before mixing with PLA had a spherical and aggregated structure (Figure 3b). The blend of sago starch and PLA is shown in Figure 3c–f, showed evident granules in the PLA compound, which was the sago starch. This granulated starch is thought to be undispersed starch in the PLA matrix. The size of the granulated starch increased as the concentration of starch in the blending system. This occurrence was evidently seen at a higher the sago starch blending with concentration more than 30%.

Figure 3 shows showed that the granulated starch was in the range of 5–10 μm at the sago with maximum concentration of 20% (SPLA10 and SPLA20). This granulated starch has a size more than 10 μm at the sago/PLA blending with more than 30% weight concentration (Figure 3d-f). These results found to be similar with Akrami et al. [21] and Salwa et al. [35] that used similar preparation method by solution mixing. However, similar morphologies also shown by a fully mechanical mixing method that reported by Taghizadeh et Favis [46] and Herrera et al. [47].

At the lower concentration of sago starch (less than 20%), the interaction between the hydroxyl group of the glucose unit in the starch and the carboxyl group of PLA was formed an interface [59]. Accordingly, at a higher concentration of more than 20%, the interface between the starch and PLA matrix is not adequate. Thus, a more pronounced phase separation is evident as depicted in Figure 3e,f.

Based on the SEM micrograph, it is found that the sago starch did not mix evenly due to differences in hydrophilicity [22, 26]. This was evidently presented at a mixing rate of 20% and above of sago starch/PLA. This immiscibility evidenced the low interface adhesion of starch particles with PLA due to the hydrophobic characteristics of PLA and hydrophilic starch [24, 60]. The sago starch/PLA mixture had a rough surface with a low level of agglomeration until phase separation occurred, when small spots and dots appeared (indicated by arrows in Figure 3c), which represent sago starch. This agrees with the findings of Wang et al. and Mueller et al. [51, 61]. Therefore, we decided to use the blend with a maximum 20 wt% starch in PLA in miniplate processing.

**Figure 4:**
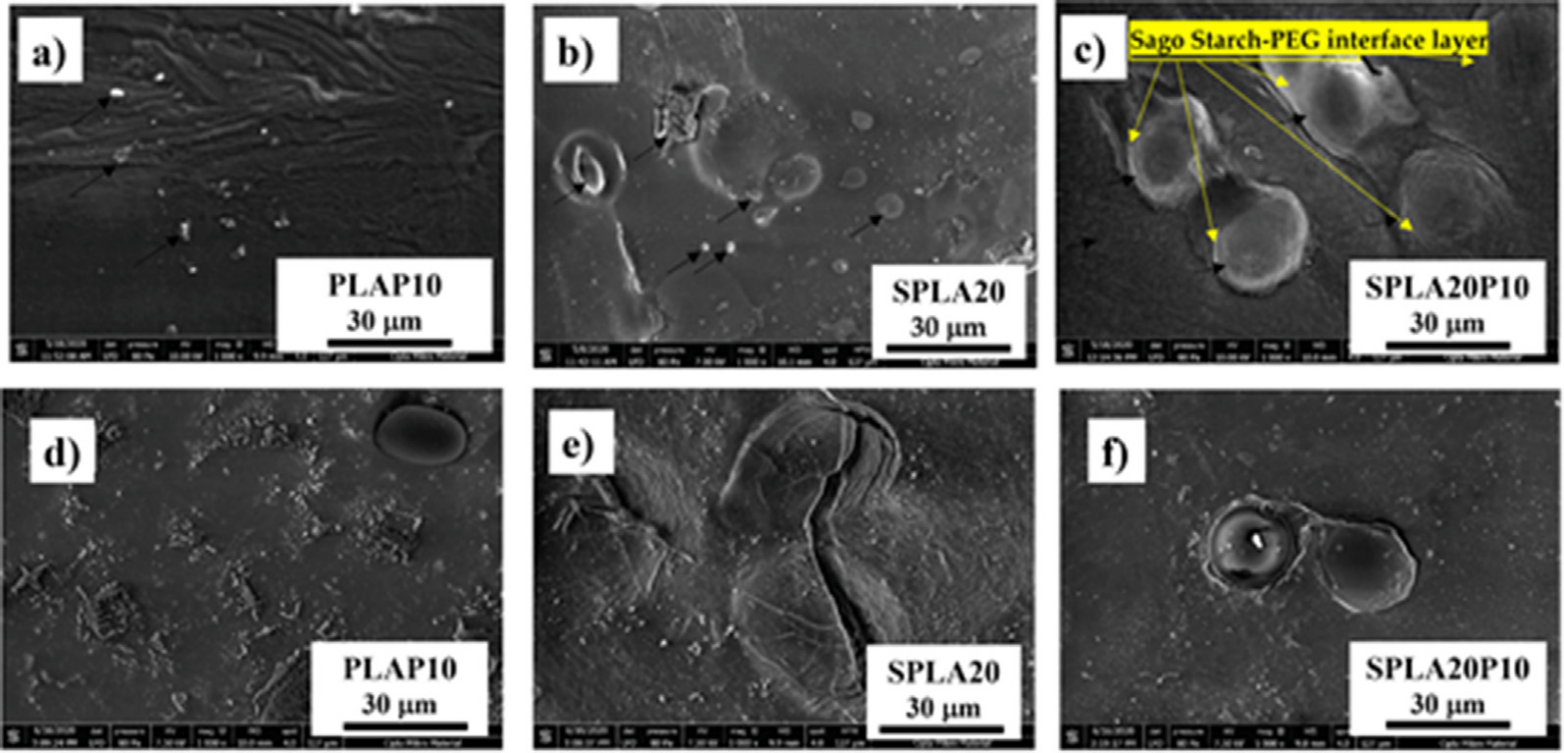
SEM images of different miniplate specimens before and after degradation: (**a**) PLA/PEG; (**b**) 20% sago starch/PLA; (**c**) sago starch/PLA/PEG; (**d**) PLA/PEG after 4 weeks' degradation; (**e**) 20% sago starch-PLA after 4 weeks' degradation; (**f**) sago starch/PLA/PEG after 4 weeks' degradation.

Significant changes in the microstructure of the miniplate were observed with the addition of PEG to the PLA matrix (Figure 4a). The PLA/PEG blend, PLAP10, had a smoother surface compared to that of pure PLA (Figure 3a). We assumed that the 10% PEG increased the processability of the PLA compound during injection molding. The spherical profile, with a lighter color on the surface, indicated the presence of starch particles that were not completely dispersed in the PLA matrix during the sago starch/PLA miniplate injection molding process (Figure 4b). The micro profile of the sago starch/PLA/PEG blend miniplate in Figure 4c shows the formation of a PEG interphase layer around the sago starch particles in the PLA matrix (as pointed by yellow line color).

Figure 4d–f shows the micrograph profiles of miniplates after 28 days of degradation testing. A rougher surface profile appeared after the immersion of the PLA/PEG miniplate specimen in the liquid environment (Figure 4d). Figure 4e depicts a micrograph of the surface of the sago starch/PLA 20% miniplate, which shows a large fracture profile after 28 days. The miniplate micrograph profile depicting the sago starch/PLA blend with the addition of 10% PEG compatibilizer is shown in Figure 4f. The addition of 10% PEG to the sago starch/PLA did not cause a surface crack, as was observed for the sago starch/PLA blend.

An additional compound, PEG, was needed as a plasticizing agent in the sago starch/PLA system to mitigate the phase separation issue. PEG forms a layer around the starch particles, which molecularly increases the interface bond between PLA and sago starch [62]. This interfacial bond of starch, PEG, and PLA matrices is caused by an increase in surface tension during the injection molding [62, 63, 64].

The micrograph of degraded SPLA20 showed a crack of dispersed starch particles on the PLA matrix after 28 days of degradation. The observation period was chosen due to the rapid growth of host bone during its remodeling in three weeks’ time [65]. This result was also evident for the sago starch/PLA/PEG blend, indicating that the interphase layer that previously formed around the starch particles (Figure 4f) was lost; thus, the outer side of the starch particles were exposed during the degradation [59]. This indicated that the interphase layer in the form of a PEG compatibilizer dispersed into the PBS solution and ultimately disappeared in the in vivo environment.

### 3.2 Thermal characteristics

**Figure 5:**
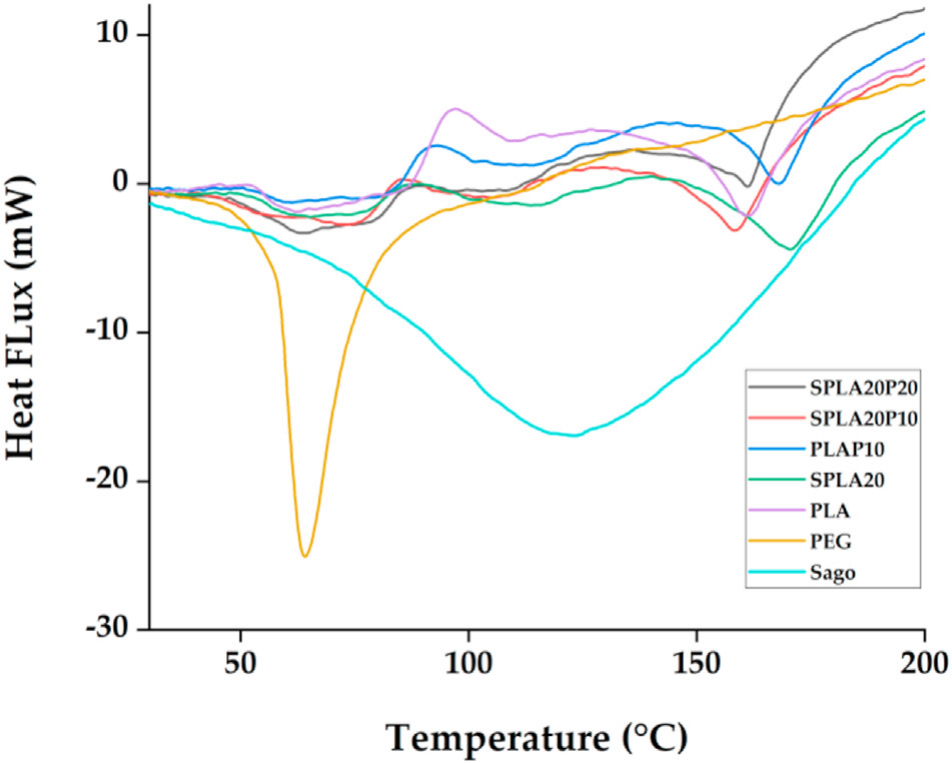
Result of thermal characterization using differential scanning calorimetry (DSC) at a heating rate of 10 °C/min.

The thermal characteristics were determined by DSC and TGA thermograms. DSC is a thermodynamical method for determining the direct heat energy uptake during a controlled increase or decrease in temperature [66]. The results of the DSC thermogram from the miniplate are shown in Figure 5. The thermogram of pure PLA had a glass transition temperature (Tg) of 51.2 °C. During the crystallization process during heating, the cold crystallization temperature (Tcc) was 96.5 °C, and the melting temperature (T_m_) was 161.2 °C. The sago starch DSC curve shows a broad endothermic peak with Tg observed at 61.3 °C. The thermal behaviour of PEG showed a sharp endothermic peak with Tm of 57.7 °C. From the observation, PEG and sago starch addition slightly decreased the thermal transition of material. PEG addition should decrease the Tg of PLA compared to the sago starch, according to Baiardo's report [67]. Other reports stated that the Tg might also increase with the existence of PEG at a certain concentration [68, 69]. Table 3 also shows that Tg of all the blended materials had T_g_ lower than pure PLA. It is beneficial for maxillofacial implants since the osteosynthesis devices must be adapted optimally to fit the bone [70]. While implants are placed on the facial bones, the implants were softened to the T_g_ to shape the facial contours.

**Table 3:** Summary of physicochemical properties of miniplate specimens produced with various material blends using pure PLA, sago starch/PLA, PLA/PEG, and sago starch/PLA/PEG. T_g_, glass transition temperature; T_cc_, cold crystallization temperature; T_m,_ melting temperature; H_cc_, enthalpy of crystallization; X_c_, crystallinity degree; T_onset_, temperature of the onset of degradation.

Sample	T_g_ (°C)	T_cc_ (°C)	T_m_ (°C)	ΔH_cc_ (J/g)	ΔH_m_ (J/g)	% X_c_	T_onset_ (°C)
PLA	51.2	96.5	161.2	9.34	12.46	-	294.9
Sago	61.3	N/A	262.9	N/A	86.52	30.15	274.7
PEG	N/A	N/A	57.7	N/A	106.40	77.9	369.3
PLAP10	50.9	85.7	154.1	5.53	7.86	11.71	300.2
SPLA20	49.2	79.2	150.5	4.09	19.24	20.87	261
SPLA20P10	41.2	80.6	144.8	3.92	17.28	22.8	261
SPLA20P20	44.8	82.8	150.9	2.51	10.56	38.92	277.3

**Figure 6:**
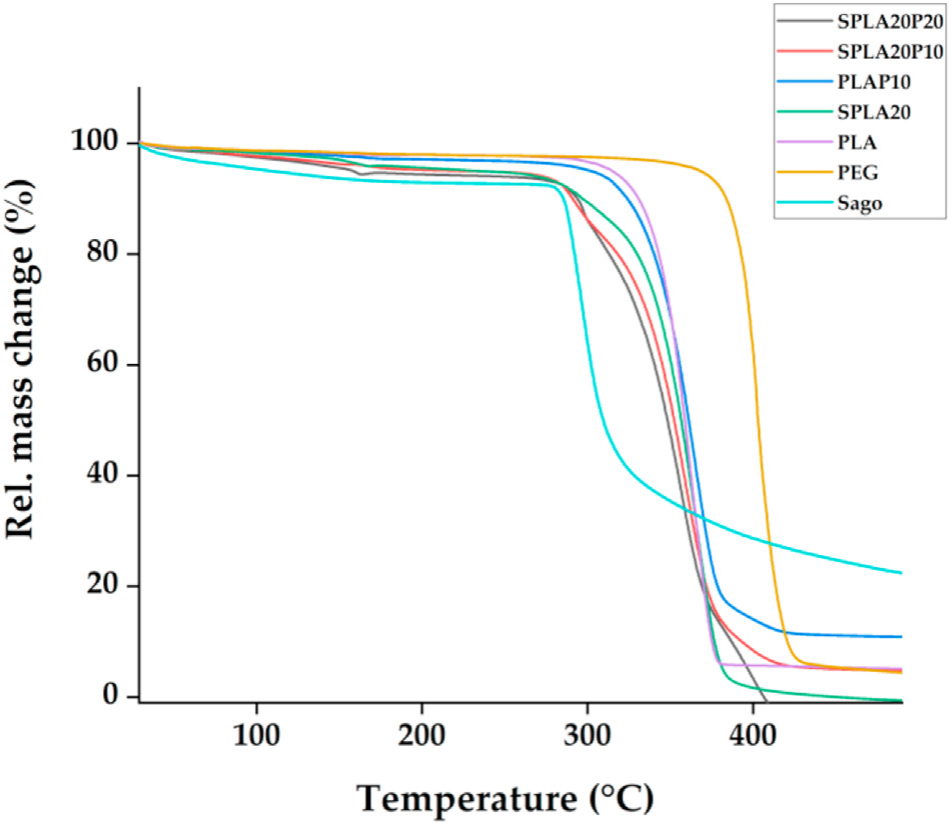
Result of thermal characterization using thermal gravimetric analysis (TGA) for pure PLA, PLA/PEG, sago starch/PLA, and sago starch/PLA/PEG compounds.

TGA analysis is an analytical method that monitors the weight change that occurs as a sample is heated at a constant rate to assess the thermal stability of the material and the fraction of volatile components [71]. Figure 6 shows the TGA observations from PLA, Sago, PEG, and blended materials. As shown, all the materials had a similar mass loss at approximately 300 °C–400 °C. It is observed that PEG had the highest T_onset_, and sago had the smallest T_onset_ compared to other materials. It is also confirming that the addition of 20% sago starch decreased T_onset_ by 33.9 °C, which is a much as 11.5% compared to pure PLA. PEG addition slightly modified the T_onset_ of the blending, as shown by the PLAP10 specimen. This was also observed for the sago starch/PLA/PEG blending system. We observed 11% and 6% lower Tonset with the addition of 10% and 20% PEG to the sago starch/PLA blend, respectively. It can be concluded that the addition of sago starch had a great influence on the thermal degradation behaviour of PLA, and the decomposition temperatures all shifted to lower temperatures compared to pure PLA. TGA analysis showed that these materials were less thermally stable compared to pure PLA. On the contrary, the addition of PEG only slightly increases the thermal degradation behavior of PLA.

### 3.3 X-ray diffraction analysis

**Figure 7:**
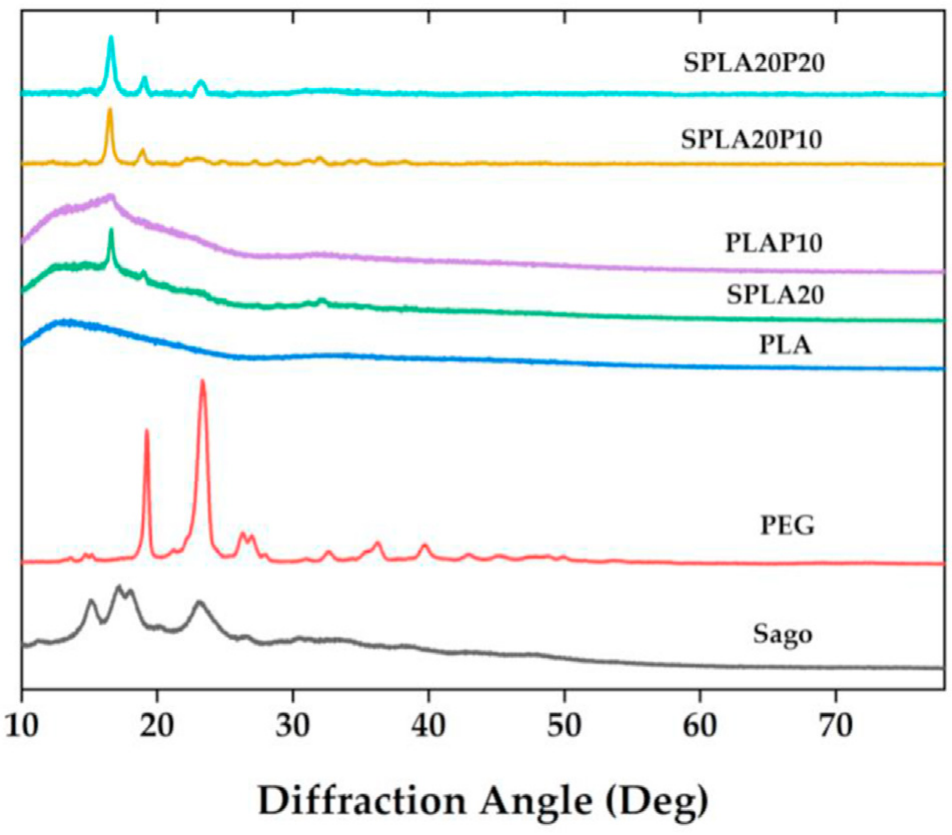
The crystalline phase and molecular interaction of the miniplate specimens and pure material (Sago starch and PEG).

The diffraction patterns indicating the crystallinity phase of PLA, sago starch/PLA, and sago starch/PLA/PEG blends are shown in Figure 7. The diffraction angle, 2θ, was presented from 5° to 80°. The XRD pattern for the PLA did not show any characteristic peak, which indicates that the structure of PLA is amorphous. Chu et al. [72] and Silverajah et al. [73] also reported the amorphous characteristics of PLA peak. The PEG, in this research, we use PEG4000, has identified two strong and sharp diffraction peaks at 19.24° and 23.3° with some other small diffraction peaks [74]. Additionally, sago starch displayed major diffraction angle of 15.06°, 17.3°, 18.04° and 23.16° that shows the characteristics of C-type crystalline patterns. Some studies already investigated the diffraction pattern of sago starch and reporting the same characteristics C-type crystalline structure for sago starch [75]. The characterization of each pure components is allowing us to identify these substances individually.

XRD patterns of sago starch/PLA and sago starch/PLA/PEG phases are also shown. The XRD pattern of PLA/PEG (PLAP10) exhibited small and less sharpness diffraction peak at around 16.4°, whereas the sago starch/PLA (SPLA20) showed small peak at 16.6°. It indicates that sago starch blending increases the crystallinity. The different compositions of blended material sago starch/PLA/PEG also showed different peaks. SPLA20P10 had a small broad diffraction peak at 16.4° while SPLA20P20 had a strong and sharp diffraction peak at 16.8°, 19.3° and 23.4°. This diffraction angle is indicated that increasing the PEG concentration in the sago starch/PLA mixture shifts the crystallinity peak of the compound.

The ratio between the area related to the crystalline phase and the overall area under the XRD curve was used to compute the crystallinity degree (%X_c_) using formula (1) [76]:

$$\%X_{c}=\frac{Area\;of\;crystalline\;peaks}{Peaks\;total\;area}\times 100\%$$

The results of crystallinity degree calculation are shown in Table 3. From the calculation, we found that pure PEG had the highest crystallinity of 77.9%, whereas pure sago starch had a crystallinity degree of 30.15%. This resulted that PEG significantly modified the crystallinity phase of the other mixing compounds. The mixture of SPLA20 had a crystallinity of 20.87%, while PLAP10 had lower crystallinity at 11.71%. The mixture of SPLA20P10 and SPLA20P20 compound had crystallinity degrees of 22.8% and 38.92%, respectively. These results show that PEG increases the crystallinity degree of materials.

The thermal characterization confirmed that the addition of PEG significantly increased the melting temperature (T_m_) and significantly decreased the glass transition temperature (T_g_). We think that the PEG contributed to increasing the crystallinity of the PLA blend with sago starch. This increasing crystallinity was also confirmed by FT-IR scanning of sago starch/PLA. Note that pure PLA was found to have a more amorphous structure [77]. A higher peak wave was observed for the specimen, which agrees with the findings of Akrami and Ferrarezi [21, 64].

### 3.4 Polymer interaction (FT-IR analysis)

**Figure 8:**
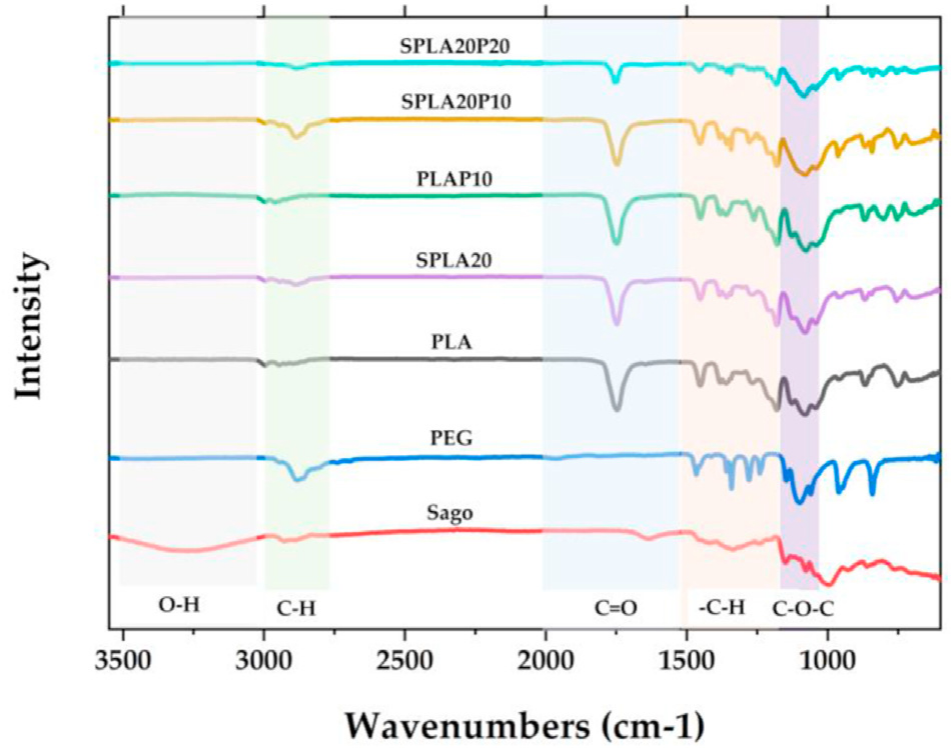
Fourier transform-infrared (FT-IR) spectra for pure PLA, sago starch, PEG and blend specimens: sago starch/PLA, PLA/PEG, and sago starch/PLA/PEG.

The interaction between polymers was observed using infrared (IR) spectroscopy, as shown in Figure 8. For the miniplate with pure PLA, we identified several absorption bands in the infrared range. We observed the infra-red (IR) spectra at 2995 and 2945 cm^−1^, which indicated to asymmetric and symmetric C–H stretching region, respectively. The strong absorption band at 1747 cm^−1^ was characterized for C=O stretching peak of ester group. The characteristics of stretching peaks at 1180 cm^−1^ indicating the presence of C–O. Strong band at 1080 of C–O–C stretching is also shown in PLA. Pure PEG recorded its C–H stretching at 2881 cm^−1^, CH_2_ scissoring at 1466 cm^−1^, strong band of C–O–C stretching at 1098 cm^−1^, –CH_2_ twisting at 960 cm^−1^, and –CH_2_ wagging at 841 cm^−1^. Pure sago starch was identified by broad peak around 3600-2995 cm^−1^ indicating O–H stretching vibrations. C–H stretching band was found at 2926 cm^−1^. Band corresponding to C–H and O–H bending were observed at 1634 and 1418 cm^−1^, respectively. Sago starch also showed a band at 1336 cm^−1^ which indicated –CH_2_ bending, bands at 1076 cm^−1^ showed C–O–C bending.

As shown in Figure 8, PLAP10 shows similar peak with pure PLA. In PLAP10, the strong peak at 1747 cm^−1^ for C=O stretching peak of ester group appeared. It is also appeared for mixing sago starch and PLA in PLAP20. The peak O–H which corresponds to hydroxyl group in sago starch does not appear in the blending. PEG/PLA, sago starch/PLA, and sago starch/PEG/PLA shows similar absorption peak as pure PLA. This means that there is no effect on the addition of sago starch on PLA blending. This ensures that the blend does not form new bonds or intense chemical interactions within the mixture, which consistent with previous result [78].

### 3.5 Mechanical properties

**Figure 9:**
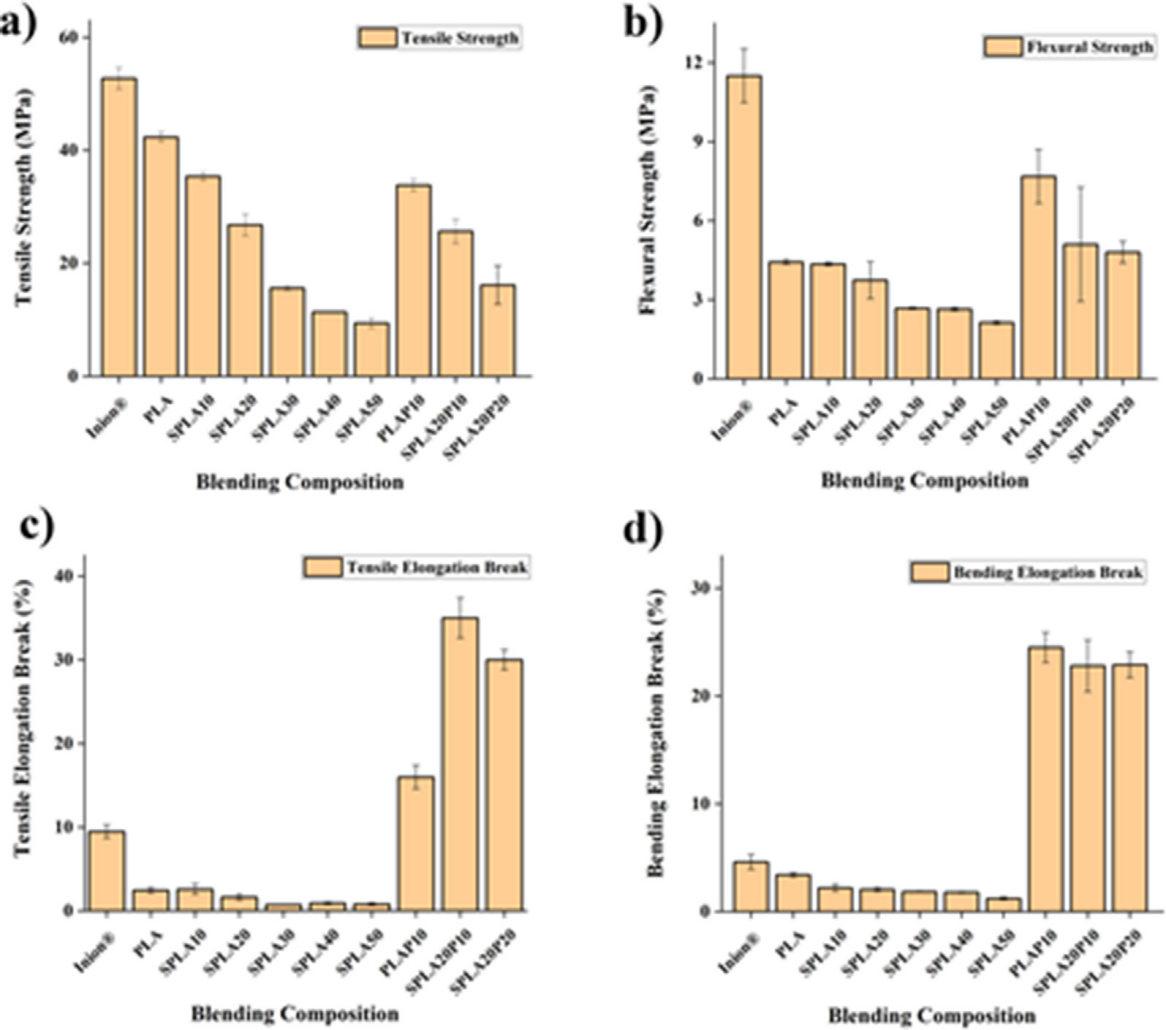
Tensile and bending properties of PLA-based miniplates: (**a**) tensile strength; (**b**) flexural strength; (**c**) tensile elongation-at-break strength and (**d**) tensile elongation-at-break strength.

Miniplate specimens were subjected to tensile and bending tests to determine tensile strength, tensile modulus, elongation break, flexural stiffness, and flexural strength. The results are summarized in Table 4. The addition of sago starch to the PLA matrix at concentrations from 10% to 50% by weight consistently reduced tensile strength, modulus, and elongation at break values. Specifically, the addition of sago starch until 20% (SPLA20) maintained the tensile strength of about 62% that of pure PLA (Figure 9a). At a sago starch content above 20%, the strength was 22%–36% that of pure PLA. The tensile strength with the addition of 10% PEG (PLAP10) was up to 80% of that of pure PLA. Meanwhile, starch/PLA/PEG blends resulted in approximately 60% and 20% of the pure PLA for SPLA20P10 and SPLA20P20, respectively. PEG also significantly increased the tensile elongation at break up from around 6-fold–20-fold compared to the sago starch/PLA blends. On the other hand, the addition of PEG could reach the bending elongation at break of 11-fold–20-fold compared to the sago starch/PLA blends. The addition of PEG plasticizers significantly reduces the tensile strength and modulus of elasticity [79]. This was confirmed by Ferrarezi and Kozlowski, who found that the addition of PEG plasticizers affects starch/PLA mixtures; the addition of plasticizers increased the drawability of the miniplate significantly but decreased the modulus of elasticity [64].

**Table 4:** Mechanical properties of miniplate specimens produced from various sago starch/PLA/PEG in blend formulations.

Sample	Tensile Strength [MPa]	Tensile Modulus [GPa]	Flexural Strength [MPa]	Flexural Modulus [GPa]	Tensile Elongated Break [%]	Bending Elongated Break [%]
Inion®	52.74 ± 1.9	2.54 ± 0.8	11.50 ± 1.0	0.89 ± 0.1	9.48 ± 0.8	4.61 ± 0.7
PLA	42.33 ± 0.8	3.56 ± 0.3	4.43 ± 0.1	0.45 ± 0.0	2.46 ± 0.3	3.41 ± 0.2
SPLA10	35.37 ± 0.7	2.42 ± 0.1	4.35 ± 0.1	0.60 ± 0.1	2.59 ± 0.6	2.19 ± 0.3
SPLA20	26.77 ± 1.9	1.96 ± 0.2	3.74 ± 0.7	0.48 ± 0.1	1.65 ± 0.4	2.05 ± 0.2
SPLA30	15.59 ± 0.4	1.35 ± 0.2	2.67 ± 0.0	0.54 ± 0.1	0.73 ± 0.0	1.85 ± 0.0
SPLA40	11.37 ± 0.1	1.53 ± 0.3	2.64 ± 0.1	0.49 ± 0.1	0.91 ± 0.1	1.78 ± 0.1
SPLA50	9.39 ± 0.8	1.35 ± 0.5	2.13 ± 0.1	0.29 ± 0.1	0.81 ± 0.2	1.21 ± 0.1
PLAP10	33.81 ± 1.0	1.60 ± 0.8	7.68 ± 1.0	0.33 ± 0.1	16.34 ± 1.5	24.51 ± 1.4
SPLA20P10	25.63 ± 2.2	1.57 ± 0.5	5.11 ± 2.2	0.28 ± 0.1	35.34 ± 2.4	22.82 ± 2.5
SPLA20P20	16.15 ± 3.4	0.87 ± 0.7	4.83 ± 0.4	0.21 ± 0.0	30.19 ± 1.2	22.94 ± 1.3

The bending test revealed a similar behavior, with the flexural strength of sago starch/PLA decreasing compared to the pure PLA (Figure 9b). The addition of PEG to the blend increased the flexural strength of the sago starch/PLA blend. However, the starch in PEG did not significantly impact the flexural modulus. The flexural modulus of sago starch/PLA/PEG decreased around 50% compared to that of the pure PLA miniplate. Similarly, PEG significantly increasing the elongation at break up to seven times (Figure 9c).

A mechanical measurement was also recorded on a commercial product with a similar design, Inion® (Inion Oy, Tampere, Finland). This product is mainly based on a mixture of PLA/PLGA with a weight percent of 85:15 [80]. The tensile and bending test revealed that the Inion® miniplate has a higher tensile strength, tensile elongation break, and flexural strength compared to our pure PLA miniplate. Only the tensile modulus of pure PLA was higher value than that of the Inion® miniplate. Similarly, the mechanical characteristics of the sago starch/PLA/PEG blend were also lower that of the Inion® plate. However, the elongation of the sago starch/PLA/PEG blend had a higher value compared to Inion® by about three-fold (Figure 9d). This longer elongation break has benefit in toughening the miniplate during the fixation.

The addition of PEG was the main contributor to the change in mechanical characteristics. When the PLA matrix deforms in the presence of pressure and increased shear forces during the injection molding process, the PEG interface forms effectively around the starch particles. Thus, the addition of a PEG compatibilizer can prevent the formation of brittle fractures and significantly increase the elongation at break of the miniplate.

The mechanical testing confirmed the poor interfacial interaction between starch and PLA, as the tensile strength was consistently reduced with the addition of higher concentrations of sago starch. Sago starch is hydrophilic, whereas PLA has hydrophobic properties [81]. The additional PEG molecular chain acts as an efficient compatibilizer in the PLA/sago starch mixture, where the PEG functions to increase the PLA and starch interface interactions, and acts as a link between molecules [59]. Thus, the addition of PEG plasticizers to the PLA/starch mixture reduced the modulus of elasticity and tensile strength but increased the elongation at break and the flexural strength.

### 3.6 Degradation characteristics

**Figure 10:**
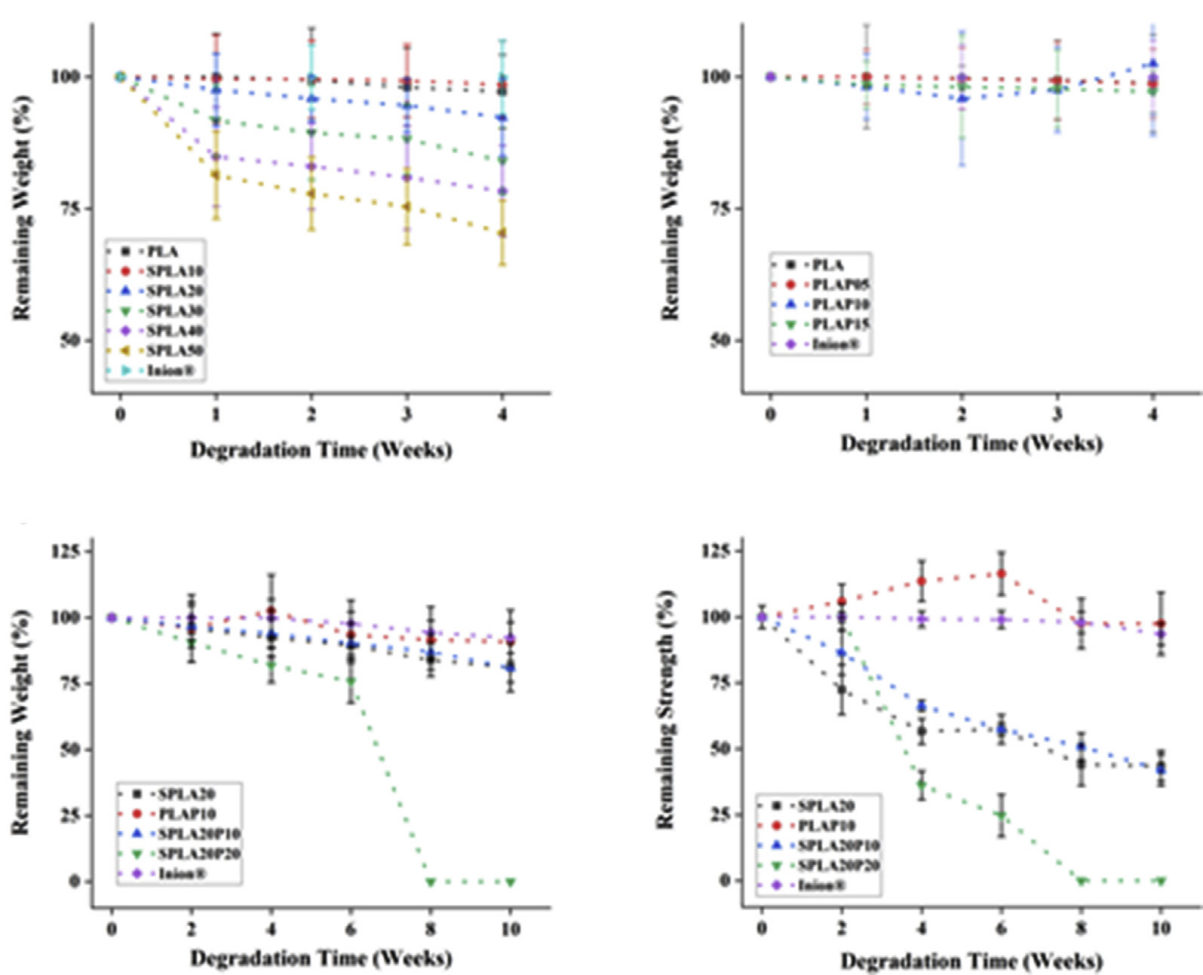
The weight loss of miniplate specimens after different degradation times in PBS solution: with various sago starch contents in PLA (10–50% sago starch); with various blend formulations of PLA/PEG, sago starch/PLA, sago starch/PLA/PEG, and a commercial miniplate.

The mass degradation of the sago starch/PLA blend in the PBS environment is depicted in Figure 10a. An increase in the sago starch content in the PLA blend significantly increased the degradation rate of the miniplate specimens. The degradation rate of miniplates with various sago starch proportions from 10% - 50% was observed to be 5%–25%, as depicted in Figure 10a. The pure PLA miniplate, the benchmark specimen, degraded at around 3%. On contrary, Figure 10b showed that a various concentration of PEG has relatively similar degradation rate. The degradation rate of PLA with 5%–15% of PEG was remain around 3%, which is relatively similar to that of commercial miniplate.

Figure 10c compares the degradation rate between the commercial product Inion® with our blended compound of sago starch/PLA/PEG in 10 weeks observation. Overall, the degradation rate of Inion® was similar to that of the PLA/PEG miniplate. The degradation rate of Inion® in this study conformed with previous studies about Inion® [82]. The sago starch specimen, SPLA20, and sago starch/PEG, SPLA20P10, have a similar degradation at around 20%. However, a bigger percentage of PEG in the specimen SPLA20P20 has a rapid degradation after 6 weeks observations and not available to be measured due to its compromised structure.

Figure 10d shows the decreasing trend of tensile strength along with the mass degradation of specimens. The commercial plate and PLAP10 had relatively strength during the 10 weeks of immersion. On the other hand, SPLA20 and SPLA20P10 declined the strength to approximately 50% after 10 weeks of immersion time. Moreover, SPLA20P20 showed a rapid decreasing of strength after its 2^nd^ week and reached a remaining of 25% remaining strength at its 6^th^ week.

**Figure 11:**
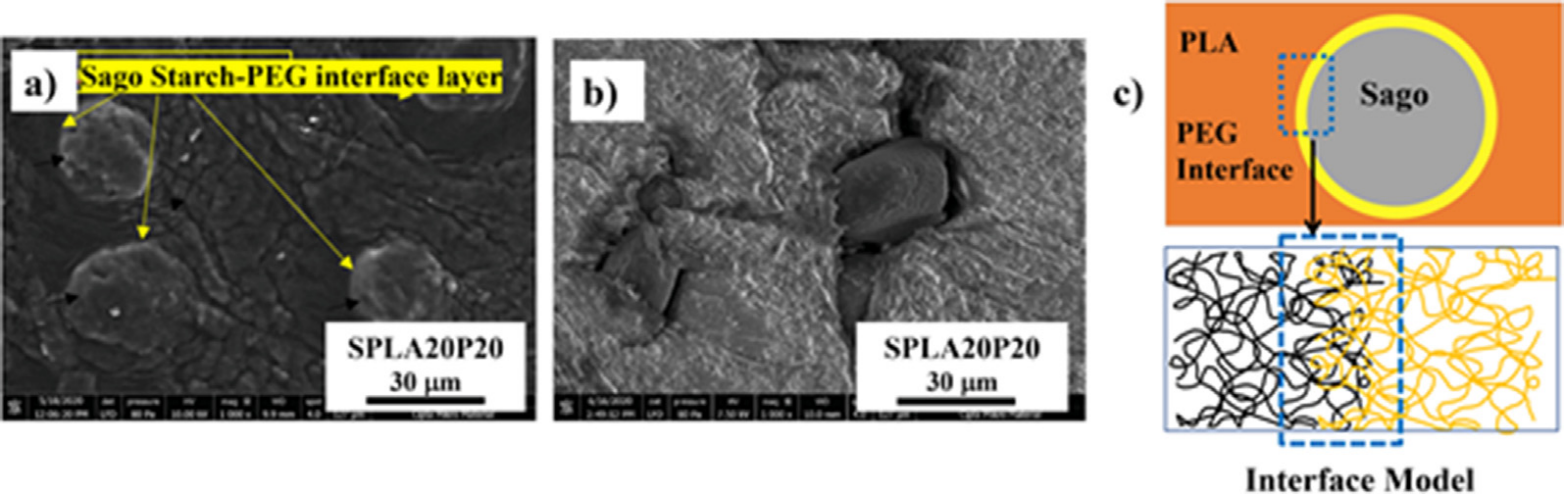
a) SEM of an aggregated sago in the PLA matrix with an interface layer of PEG in the SPLA20P20 blend system; b) a clear separable phase between sago starch and PLA at the 4^th^ week of degradation study; c) A model explained the PEG interface formed between sago and PLA layers.

The degradation rate of the miniplate specimens showed a strong relation with the structural bond between sago starch/PLA and PEG. A higher degradation rate was observed for larger fractions of the hydrophilic part due to the use of sago starch. Generally, the PEG interphase layer will increase the interface between PLA and sago starch. This interface layer was confirmed in the SEM results of the miniplate with PEG content of 10% by weight (SPLA20P10) as shown in Figure 4c. A sketch was depicted in Figure 11 to show the separable phase of sago and PLA although the PEG interface was present. It also showed that the surface of a higher PEG content in starch/PLA (SPLA20P20) has rougher surface compared to that SPLA20P10 (Figure 11a). The SEM observation in Figure 11b shown that the PEG diffused out thus make an evident crack. This phenomenon explained the loss of strength after the 6 weeks of immersion in the PBS environment that caused by the separable phase of sago and PLA matrix. Figure 11c resumed a model on how the PEG interface was formed between the two phases: sago starch and PLA.

The degradation study suggested that to gain a fast degradation, the content of sago starch was the key component. However, the study also showed that the mechanical properties were decreasing with more content of sago content. Therefore, the SPLA20 was selected as the base formulation to be mixed with PLA/PEG compound. Here, the PEG with 10% concentration in the PLA, PLAP10, showed a similar degradation with other PLA/PEG content (Figure 10b). However, our study showed that the more PLA content might decrease the strength further. The mixing of the two blending systems formed a ternary composition, SPLA20P10, that shown to have the stronger sago starch/PLA bonding. The remaining strength of SPLA20P10 shown to be higher than the SPLA20 and SPLA20P20. Note that, the strength was aimed to be kept at least 70% of its initial strength at the 4^th^ week of immersion time. In this case, the SPLA20P10 found to meet the miniplate requirement in term of strength and degradation rate.

### 3.7 Cell viability result

The effect of starch and PEG composites toward proliferation of human stem cell line was evaluated by using the MTS assays. The PLA miniplate and the sago starch/PLA has a lower viability at around 55 ± 11%, whereas the PLA/PEG and starch/PLA/PEG miniplate has a viability around 60%–70% after 24 h of cell culture (Figure 11a). After 48 h, the starch based miniplate, sago/PLA and starch/PLA/PEG showed a higher viability compared to the PLA and PLA/PEG miniplate. The PLA has an increasing of cell viability at around 73% after 72 h of cell culture, whereas the PLA/PEG reached at 85%. The starch/PLA and starch/PLA/PEG resulted a similar range and reached 100% cell viability.

The PLA miniplate consistently showed a relatively lowest cell viability, among the group during the 3 days observations. Note that, this relatively lowest cell viability of pure PLA has been studied to be compatible as our previous study [83]. Importantly, we showed that all composites were more compatible toward cell growth compared to that pure PLA miniplate.

**Figure 12:**
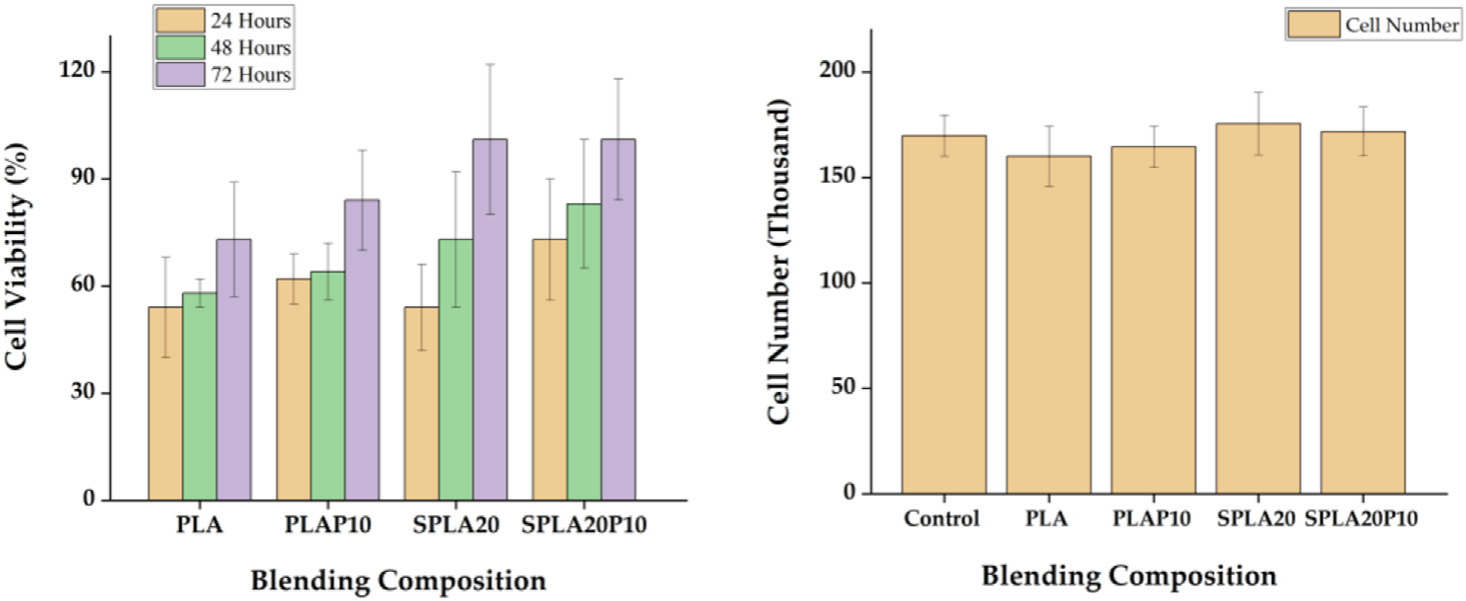
Cell viability of human stem cell when cultivated on miniplates with different blending composition.

Figure 12b performs the calculations of the number of living cells using the counting chamber method on the hemacytometer. The number of living cells in the well tissue culture plate control was used as a comparison against the number of living cells on the well tissue culture plate which was exposed directly to implants.

**Figure 13:**
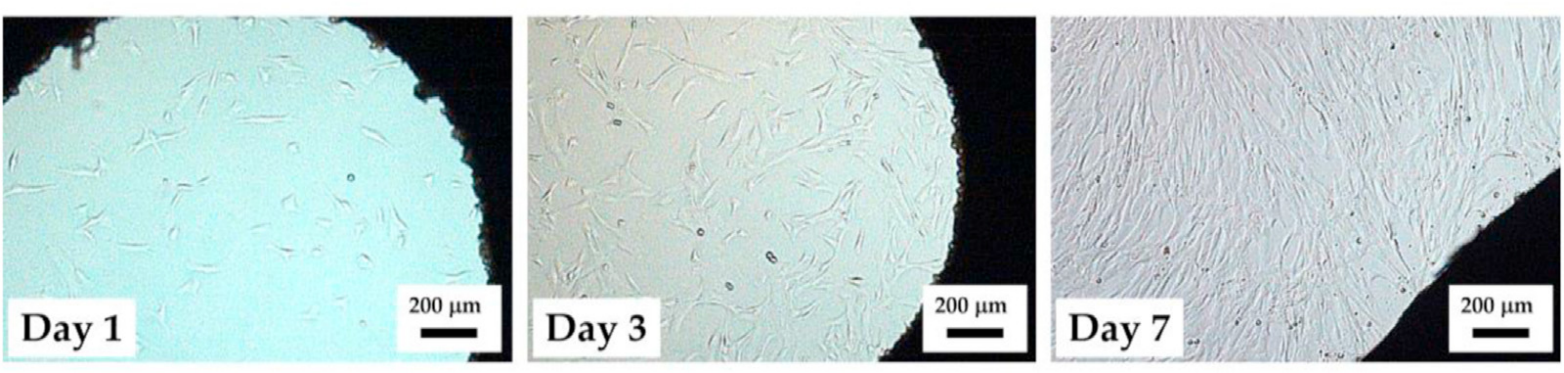
Cell viability of human stem cell when cultivated on sago starch miniplates at different time observation which were 1-3-7 days.

The following Figure 13 shows the sago starch miniplate implant in the medium for 1-, 3- and 7-days cell observations. Figure 13 also shows the shape of cells starting to proliferate in the complete growth medium as seen using an inverted microscope. This proliferation is marked by the initiation of changes in the shape of the elongated cells to be able to multiply and form a tissue that is suitable for the growth medium. The cells are denser as the longer of the culture days.

**Figure 14:**
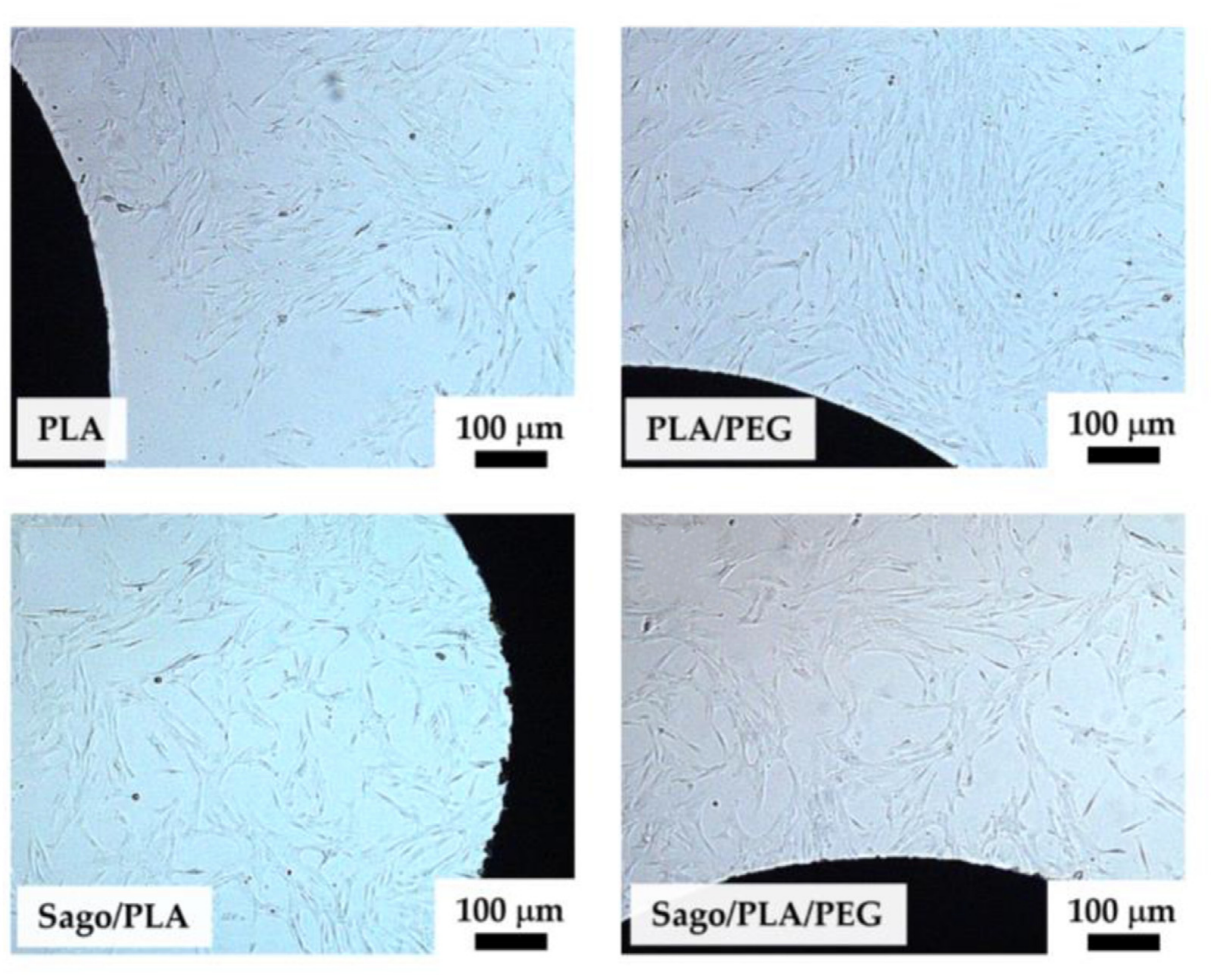
Cell viability of human stem cell when cultivated on miniplates with different blending composition.

Figure 14 shows a better magnification of the attached cells on the multiwell substrate after 72 h observation for different specimens. It can be shown that the cells were at good attachment in the medium of all specimens. Moreover, Figure 14 also depicted that there are no significant different in the cell morphologies in the substrates. This figure also confirms that the quantification was also the same trough all miniplate medium as calculated showed in Figure 12b.

The biocompatibility study confirmed that the sago starch and PEG blending with PLA have positive result in in 48 h cell culture. A significant difference between the cell viability on the PLA/PEG and sago starch/PLA/PEG miniplates were not found. On the other hand, the PLA pure miniplate shows a lower viability value. This finding agreed with the previous finding that starch has a high biocompatible performance [20]. Additionally, the PEG also found to promote the cell viability that confirmed the report of Chan et al. [84].

### 3.8 FEA results

**Figure 15:**
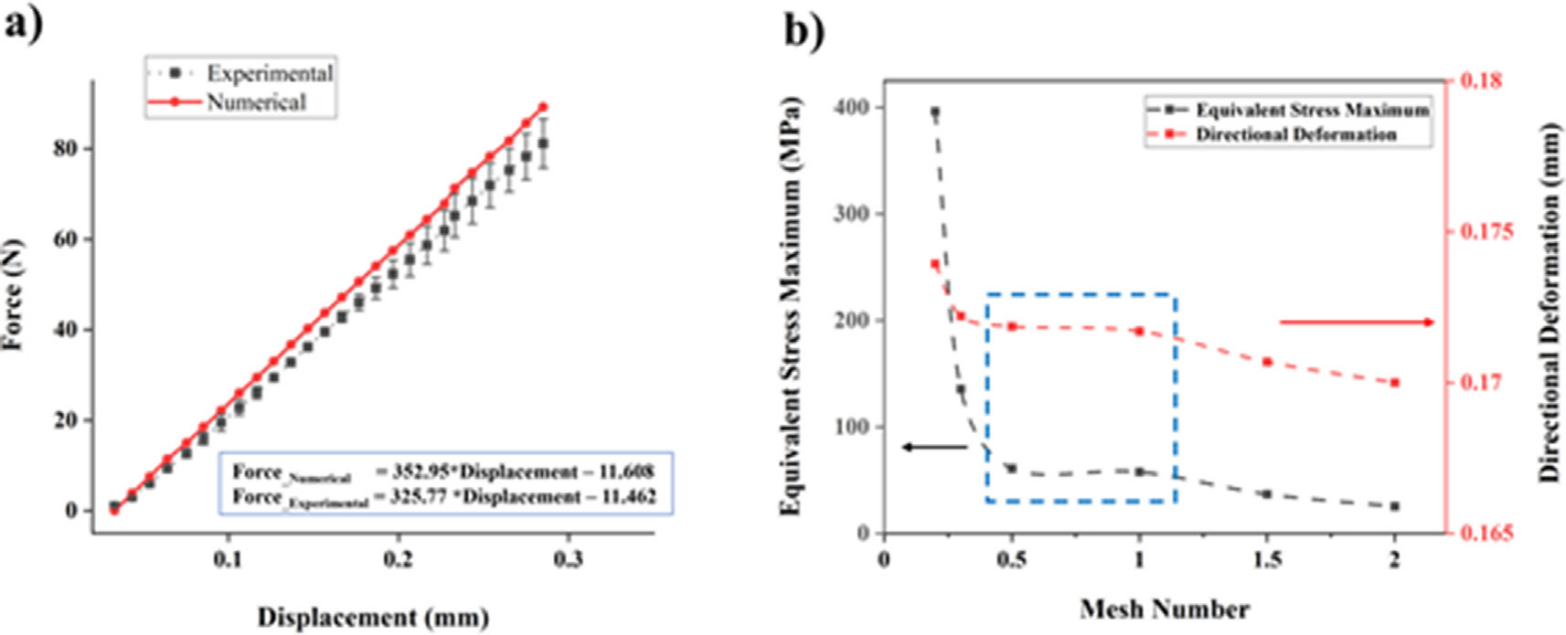
a) Comparison of the FEM simulation to replicate the experimental tensile test result and b) the grid independence test result to show the appropriate mesh size to be used in the FEA study.

Firstly, the FEA study was verified with the experimental results using the same geometrical and measured mechanical properties. The property was obtained from the material testing results (Table 4). A force–displacement curves were compared between the FEA result and the experiment (Figure 15a). The numerical (FEA) result is indicated as the red line, whereas the experimental result indicated as doted points with standard deviation values. Figure 15a also shows a linear trendline of the FEA and experimental results until the displacement of 0.3 mm. The numerical result showed a relatively higher ratio of the force to the displacement. This ratio is represented elastic modulus once it is converted to the slope at the stress and strain curve. The calculation of the slopes depicted that the numerical simulation has around 6% higher than the experimental values. However, the FEA result showed a plausible trend with the result of experimental tensile test.

Figure 15b shows the result of grid independence test to show the mesh convergence during the FEA study. Two simulated parameters were shown which were the equivalent stress and the deformation at various mesh size. The parameters have a similar profile along the mesh size of 0.2 mm until 2 mm. The profile shows a plateau line between 0.5 mm -1 mm mesh size indicated in the blue dashed box. This suggests that the use of mesh size of around 0.5 mm–1 mm shall not affect the simulation results. The mesh size of less than 0.3 mm and more than 1 mm showed a relatively significant calculation result. Therefore, a mesh size of 0.5 mm was consistently used in this work.

**Figure 16:**
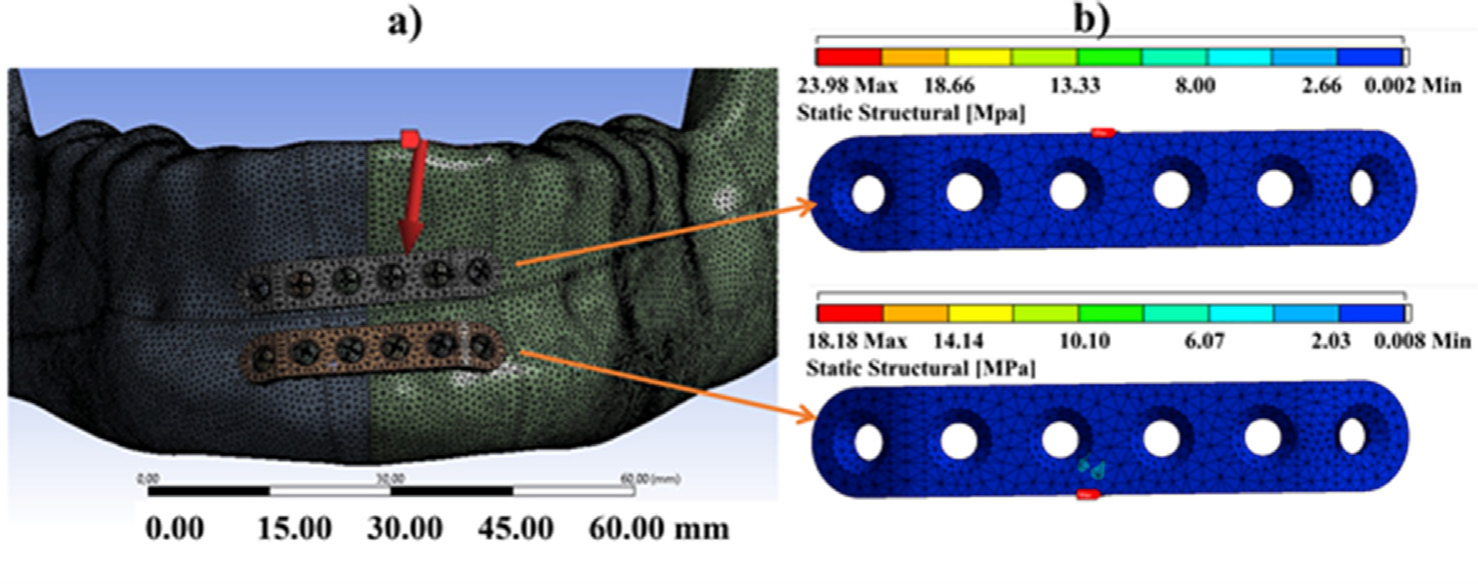
Loading simulation for SPLA20P10 miniplate when a force of 50 N was applied [51]: (**a**) the mesh result of two parallel miniplates in superior and inferior mandibular parts; (**b**) the maximum peak von Misses stresses (PVMS) from both the superior and inferior miniplates.

FEA was performed using a static structural mechanics module after assembling the mandible model and two miniplates, as shown in Figure 16. Similarly, the miniplates were assumed as a composite of an isotropic elastic material and assigned with the property of a blend of sago starch/PLA/PEG (SPLA20P10). Based on the conducted mechanical characterization, the SPLA20P10 miniplate has an elastic modulus and ultimate tensile strength of 1.57 ± 0.5 GPa and 25.63 ± 2.2 MPa, respectively. The Poisson's ratio of the implant material was 0.46, which is categorized as a high stiffness of polymer material. Figure 16 shows the loading force assumed to be exist after the post-operation on the patient. Various report shown that the load might varies from 0-100 N that trigged by the biting force. However, this study using a moderate value of 50 N that assumed to be plausible in the clinical field. Note that, most patient are not using the full biting force in early day after the operation until the bone union.

Briefly, the FEA study provide a failure criterion whenever the resulted stress exceeds a certain critical value. In this case, the miniplate is said to begin to be compromised when the von Mises stress reaches a critical value known as yield strength. Here, the von Mises stress is used to predicting the yield level of the material under the loading conditions of simple uniaxial tensile test results.

The peak von Mises stress (PVMS) values were 23.9 MPa and 18.1 MPa for the superior and inferior plates, respectively. The PVMS result of the inferior miniplate indicated that the working force was still tolerable by the material since the PVMS values to the superior and inferior plates were lower than the tensile stress of the blended system (25.63 ± 2.2 MPa).

## 4 Conclusions

This paper introduced a sago starch/PLA/PEG blend system that later served as medical degradable implant. The implant was aimed to address a mandibular fracture that required a faster degradation rate compared to available product. Ideally, a Moreover, the implant must withstand a standard load as detailed in the numerical study. Combining a fast degradability of sago starch and mechanical strength of PLA polymer was the basic idea that driven this study. Our findings agree with those of similar report that the sago starch/PLA blend had a lower tensile strength and modulus of elasticity with increasing concentration of starch. On the other hand, the PEG that added brought a higher elongation break and flexural strength that compensated the lowering of mechanical strength. Therefore, the numerical study showed that the blend formulation of 20% sago starch/PLA with 10% of PEG/PLA performed at an acceptance level of mechanical characteristics. The degradation rate of this blend system also shows a 20% higher rate compared to the available product that confirmed in 10 weeks observation. The remaining strength was achieved at around 70% of its initial value at week 4 to week 6 as expected. It was reported that the bone union was evident in that period so that the bone regains the strength to withstand the load after operation. Ultimately, the biocompatibility study showed that the sago starch and PEG in PLA compound promoted a better cell viability. This finding showed a promising result in term of its mechanical property and degradation rate in in-vitro setting. A further study shall be needed to explore the application of the miniplate in animal bone modeling.

## 5 Featured application

The work aims to introduce a new bone implant based on bio-based material namely sago starch that could contribute the degradability feature for pediatric patient with relatively fast bone growth.

## Declarations

### Author contribution statement

Y. Whulanza: Conceived and designed the experiments; Analyzed and interpreted the data; Wrote the paper.

A. Azadi: Conceived and designed the experiments; Performed the experiments; Wrote the paper.

S. Supriadi: Contributed reagents, materials, analysis tools or data.

S. F. Rahman: Analyzed and interpreted the data.

M. Chalid: Analyzed and interpreted the data; Contributed reagents, materials, analysis tools or data.

M. Irsyad: Performed the experiments.

M. H. Nadhif: Performed the experiments; Analyzed and interpreted the data; Wrote the paper.

P. Kreshanti: Conceived and designed the experiments.

### Funding statement

This work was supported by the LPDP Ministry of Finance Republic of Indonesia under grant Rispro Invitasi 2019 (no. UI SK KEP-52/LPDP/2019).

### Data availability statement

Data included in article/supp. material/referenced in article.

### Declaration of interests statement

The authors declare no conflict of interest.

### Additional information

No additional information is available for this paper.
